# Supplementary material for: DNA-delivered antibody cocktail exhibits improved pharmacokinetics and confers prophylactic protection against SARS-CoV-2
Source: Nat Commun. 2022 Oct 6;13:5886. doi: 10.1038/s41467-022-33309-6 (PMC9537531; doi:10.1038/s41467-022-33309-6)
Supplement: Supplementary file 1 — Supplementary Information [file 41467_2022_33309_MOESM1_ESM.pdf]

DNA-delivered antibody cocktail exhibits improved pharmacokinetics and confers prophylactic protection against SARS-CoV-2

Elizabeth M. Parzych[1], Jianqiu Du[2,9], Ali R. Ali[1,9], Katherine Schultheis[3,9], Drew Frase[1,9], Trevor R. F. Smith[3,9], Jiayan Cui[2], Neethu Chokkalingam[1], Nicholas J. Tursi[1], Viviane M. Andrade[3], Bryce M. Warner[4], Ebony N. Gary[1], Yue Li[1], Jihae Choi[1], Jillian Eisenhauer[1], Igor Maricic[3], Abhijeet Kulkarni[1], Jacqueline D. Chu[1], Gabrielle Villafana[1], Kim Rosenthal[5], Kuishu Ren[5], Joseph R. Francica[5], Sarah K. Wootton[6], Pablo Tebas[7], Darwyn Kobasa[4,8], Kate E. Broderick[3], Jean D. Boyer[3],10, Mark T. Esser[5,10], Jesper Pallesen[2,10], Dan W. Kulp[1,10], Ami Patel[1,10] & David B. Weiner[1,10*]

SUPPLEMENTARY INFORMATION:

Supplementary Fig. 1. mAb characteristics and construct designs for selected SARS-CoV-2 DMAbs 2196, 2130 and 2381.

Supplementary Fig. 2. Dual plasmid systems enhance *in vitro* DMAb expression relative to single construct approaches while maintaining neutralizing activity.

Supplementary Fig. 3. Antiviral activity of 2196_FcWT and 2130_FcWT DMAbs against SARS-CoV-2 viral variants (*related to Fig. 2*).

Supplementary Fig. 4. Evaluation of wildtype (WT) DMAbs 2196, 2130 and 2381 in a non-lethal AAV6.2FF-hACE-2-transduced murine challenge model.

Supplementary Fig. 5. *In vitro* expression and validation of 2130- and 2196-based DMAbs with Fc-modified framework for extended *in vivo* half-life (YTE variants).

Supplementary Fig. 6. Prophylactic delivery of DMAb or rIgG cocktails prevents lung pathology following SARS-CoV-2 challenge in K-18 mice (*related to Fig. 4e*).

Supplementary Fig. 7. Cryo-EM data processing workflow.

Supplementary Fig. 8. Distance between neighboring dFabs in 2130 and 2196 DMAb complex.

Supplementary Fig. 9. Predictive modeling against B.1.617.2 (Delta) and B.1.1.529/BA.1 (Omicron).

Supplementary Table 1. Cryo-EM data processing specifications**.**

a

Phase	mAb Clone	Ab Family IgVH IgVL	mAb Class	Epitope	RBD Conformation	Block ACE-2?	Ref.
**EUA**	2196	IGHV1-58 IGKV3-20	I	455-456, 475-479, 484-489, 493 **<u>486, 487</u>**	Up/ Out	Yes	*Zost et al*. Nature. (2020) *Dong et al*. Nat. Micro. (2021)
**EUA**	2130	IGHV3-15/ IGKV4-01	III	345-346, 439-441, 443-447, 449-450, 452, 484, 490, 492 -494 **<u>444, 447</u>**	Up/ Out + Down/ In	Yes	

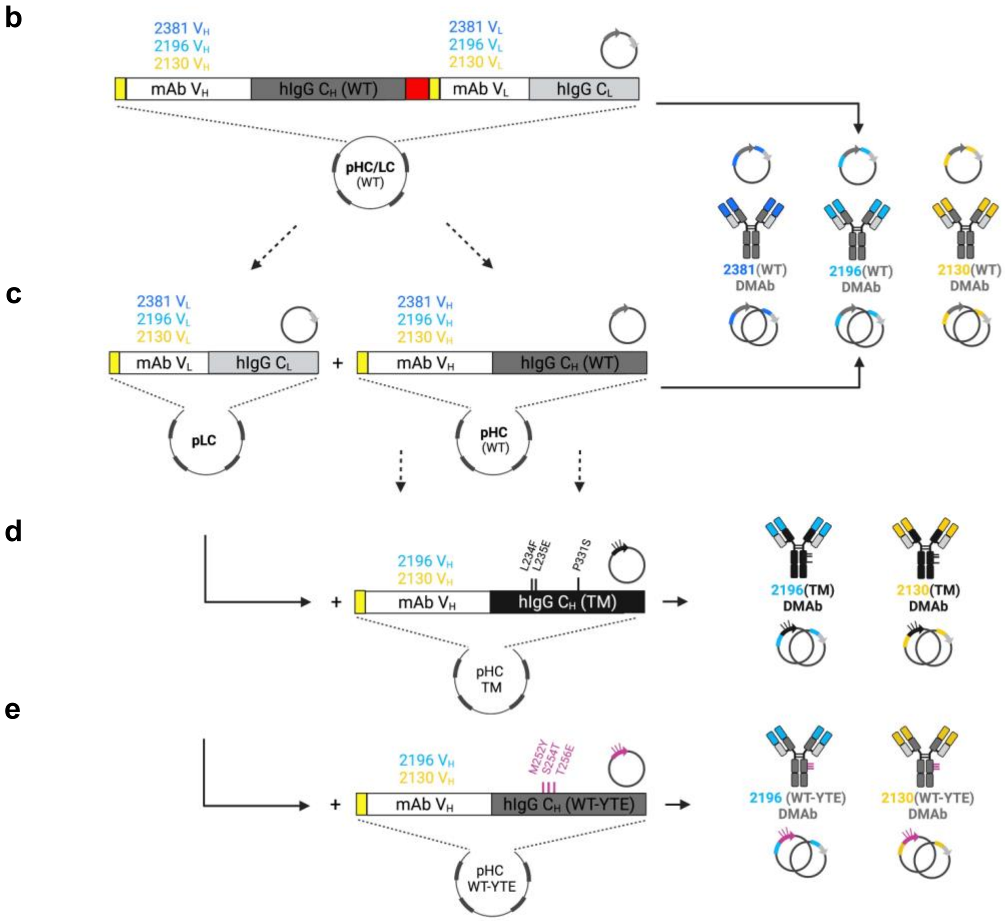

Supplementary Fig. 1. mAb characteristics and construct designs for selected SARS-CoV-2 DMAbs 2196, 2130 and 2381. a Characteristics of mAbs COV2-2196 (PDB: 7L7D) and COV2-2130 (PDB: 7L7E). **b-c** Plasmid design encoding wildtype human IgG1 (hIgG1) DMAbs. The variable heavy (V_H) and light (V_L) domains of mAbs COV2-2196, COV2-2130 and COV2-2381 were grafted onto wildtype (WT) human IgG1 constant domain framework (C_H and C_L, respectively; allotype G1m1) using **b** single plasmid (pHC/LC) or **c** dual plasmid (pLC + pHC_WT) approaches. **d** Modified pHCs constructs containing Fc mutations L234F, L235E and P331S (pHC_TM) to ablate effector functions. **e** Modified pHCs containing Fc mutations M252Y, S254T, T256E for *in vivo* half-life extension. Constructs in **c-d** were also generated using the G1m3 ("(m3)") allotype of hIgG1 as the framework. Yellow = leader sequences; red = flexible linker connecting Ab chains in single plasmid design.

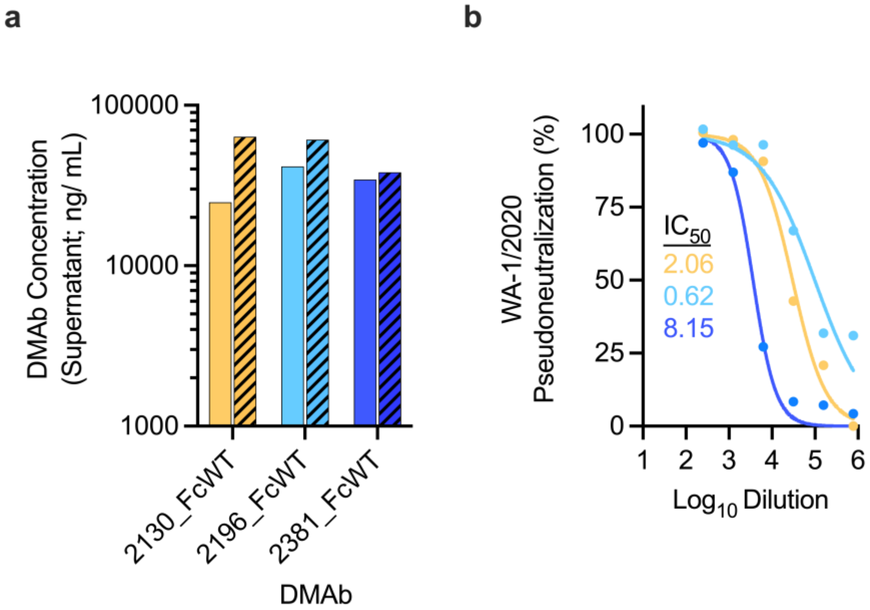

Supplementary Fig. 2. Dual plasmid systems enhance *in vitro* DMAb expression relative to single construct approaches while maintaining neutralizing activity. Parallel *in vitro* expression and evaluation of single and dual plasmid systems was performed for the indicated DMAbs. **a** DMAb quantification using an anti-human IgG ELISA following single (solid bars) or dual plasmid (hatched bars) delivery; bars represent the average titer of transfection duplicates for each construct. **b** Neutralizing activity of dual plasmid constructs against SARS-CoV-2 pseudovirus (USA-WA/2020); best-fit lines and individual data points (average derived from technical replicates) are displayed. Calculated IC50s are shown. Source data are provided as a Source Data file.

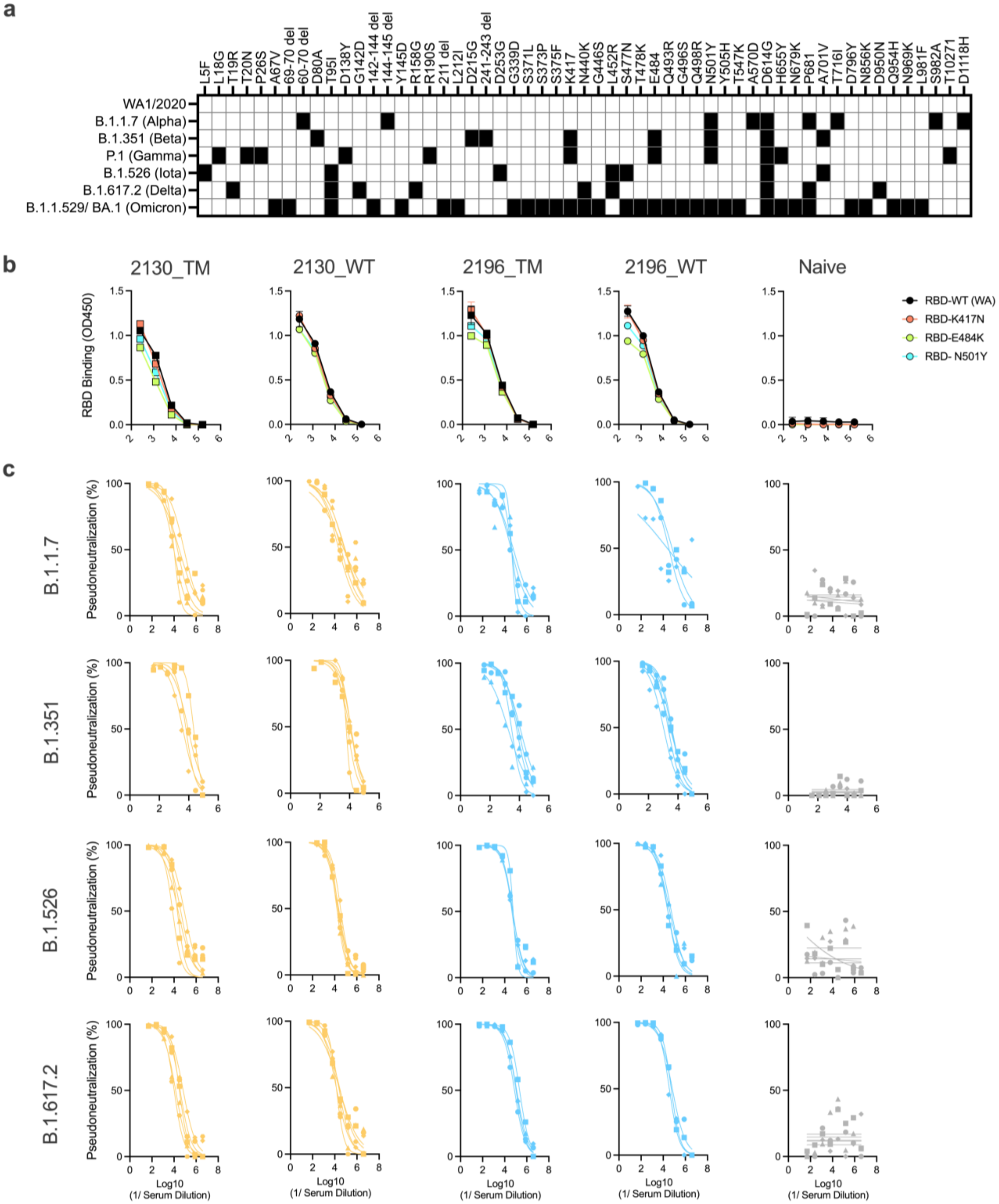

Supplementary Fig. 3. Antiviral activity of 2196_WT and 2130_WT DMAbs against SARS-CoV-2 viral variants (*related to Fig. 2*). a Summary of mutations identified throughout the SARS-CoV-2 S protein from the indicated variants. **b** Relative binding of sera pools (1 sample/construct) containing the indicated *in vivo*-launched DMAbs to various mutant RBDs via ELISA. Naïve serum was used as a control. Graphs depict the binding curves (OD450) for each pool (average derived from technical replicates). **c** Neutralizing activity of sera from animals (n = 3-5 independent biological replicates, as in *Fig. 2*) administered the indicated DMAb(s) against SARS-CoV-2 (WA1/2020), B.1.1.7, B.1.351, B.1.526 and B.1.617.2 pseudoviruses. Neutralization curves of individual serum samples (best-fit lines and individual data points derived from technical replicates) displayed for each serum sample. Matched ID50s against each variant compared to WA1/2020 are shown in *Fig. 2*. Binding and neutralization data are representative of >2 independent experiments. Source data are provided as a Source Data file.

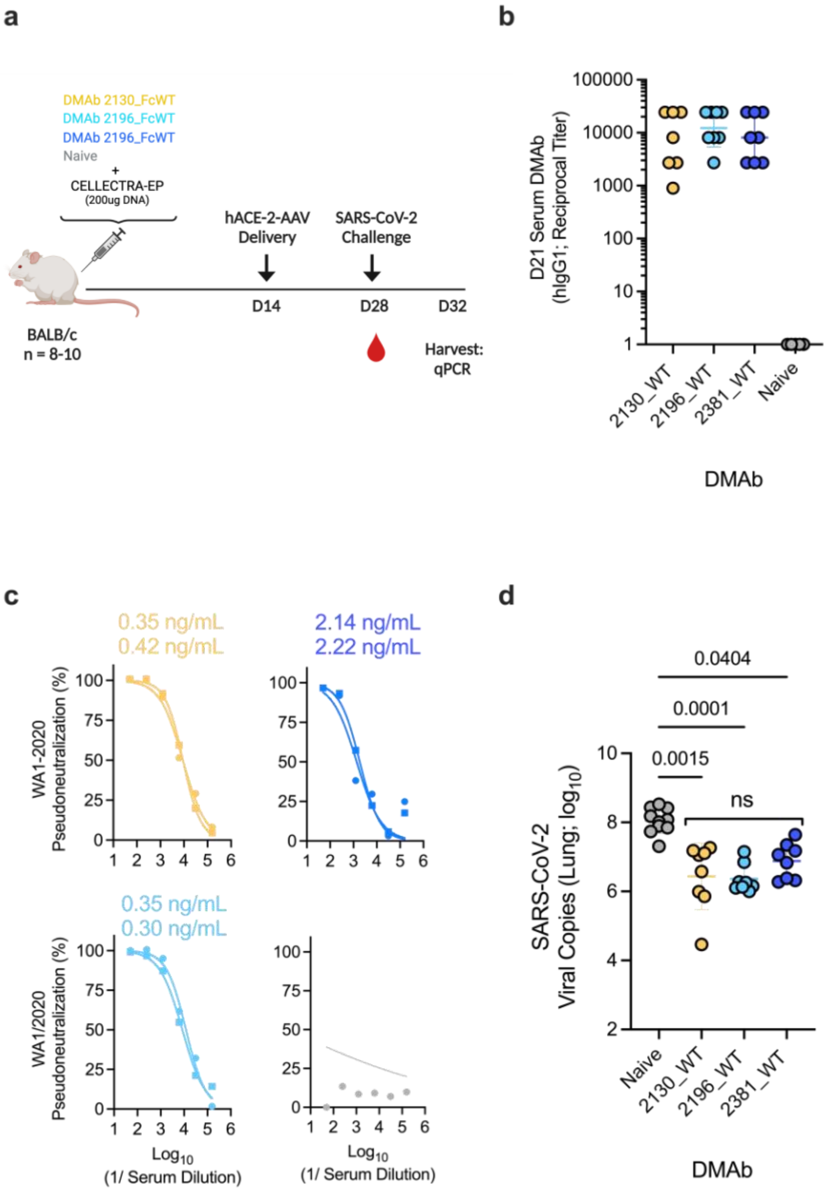

Supplementary Fig. 4. Evaluation of wildtype (WT) DMAbs 2196, 2130 and 2381 in a non-lethal AAV6.2FF-hACE-2-transduced murine challenge model. a Schematic of efficacy study conducted in 6-8wk old female BALB/c mice using an AAV-ACE2 challenge model (n = 8 (DMAbs) or 10 (naïve) independent biological replicates)**. b** Endpoint titers in the sera of DMAb-treated mice at the time of harvest (D4 post-challenge; GM +/- GSD shown). **c** Neutralization activity of *in vivo*-launched DMAbs against pseudotyped SARS-CoV-2 (USA-WA1/2020); curves (best-fit lines and individual data points derived from technical replicates) of two representative serum samples per group are shown. Calculated IC50s are depicted. Naïve sera served as a control**. d** Viral load (copies/ g) in the lungs of DMAb-treated and control mice at D4 post-challenge as determined by qPCR (GM (+/- GSD)). Group differences determined by Kruskal-Wallis test followed by Dunn's post hoc analysis. *P*-values indicated. Source data are provided as a Source Data file.

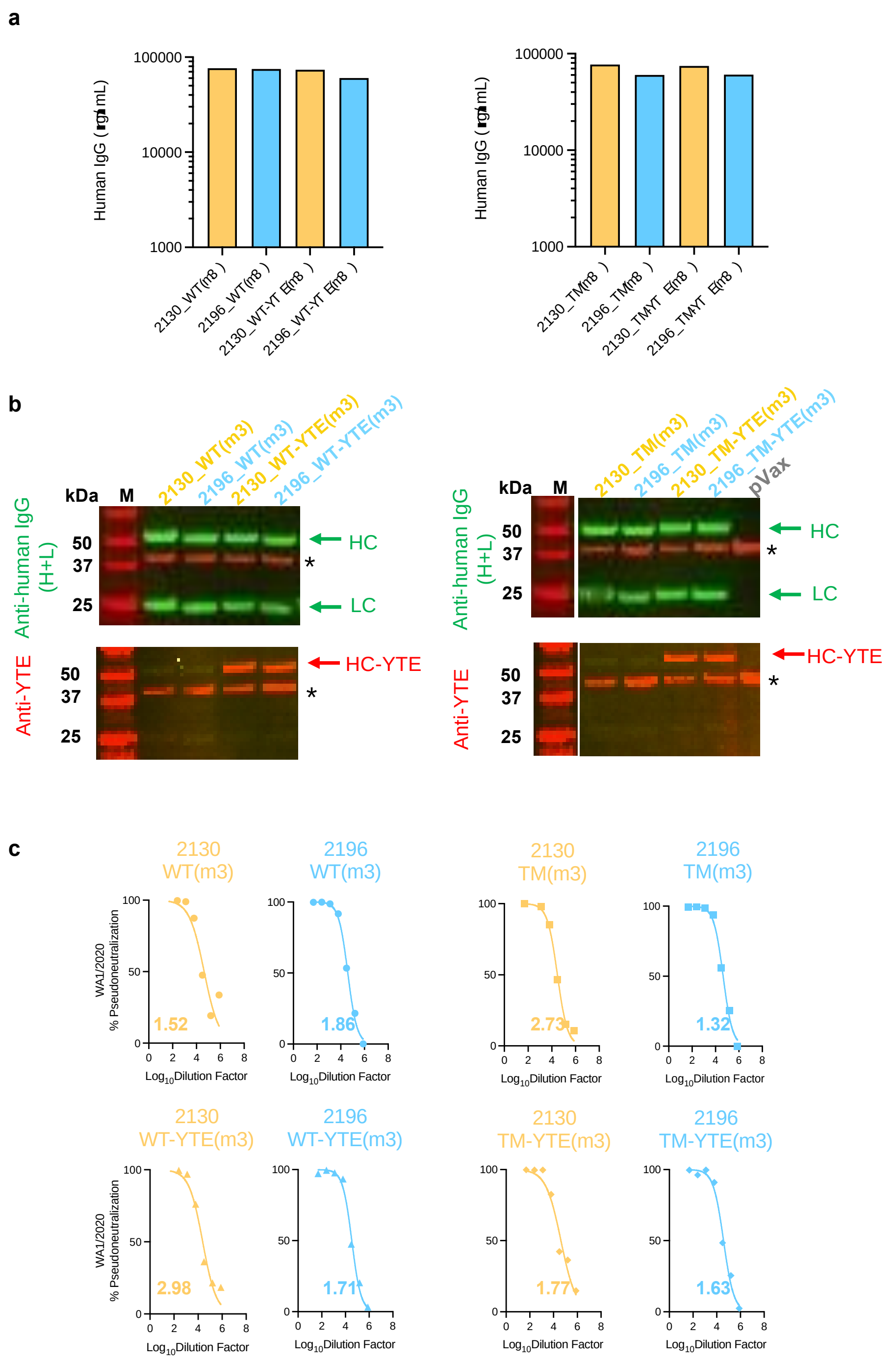

Supplementary Fig. 5. ***In vitro*** **expression and validation of 2130- and 2196-based DMAbs with Fc-modified framework for extended** ***in vivo*** **half-life (YTE variants). a-c** Analysis of the indicated DMAbs following *in vitro* expression**. a** DMAb quantification in culture supernatant; graph displays the average titer (derived from transfection duplicates) for each construct**. b** Supernatants (1 sample/construct) containing the indicated DMAbs (200 ng/lane) were analyzed via western blot using anti-human IgG (h+l)-HRP (top blots) or anti-YTE IgG (bottom blots). Beta actin was visualized on all blots as a loading control (asterisks); LC = light chain, HC = heavy chain. **c** Neutralizing activity (best-fit lines and individual data points derived from technical replicates) of the indicated DMAb sample against SARS-CoV-2 (USA-WA1/2020) pseudovirus. Calculated IC50s are displayed. Source data are provided as a Source Data file.

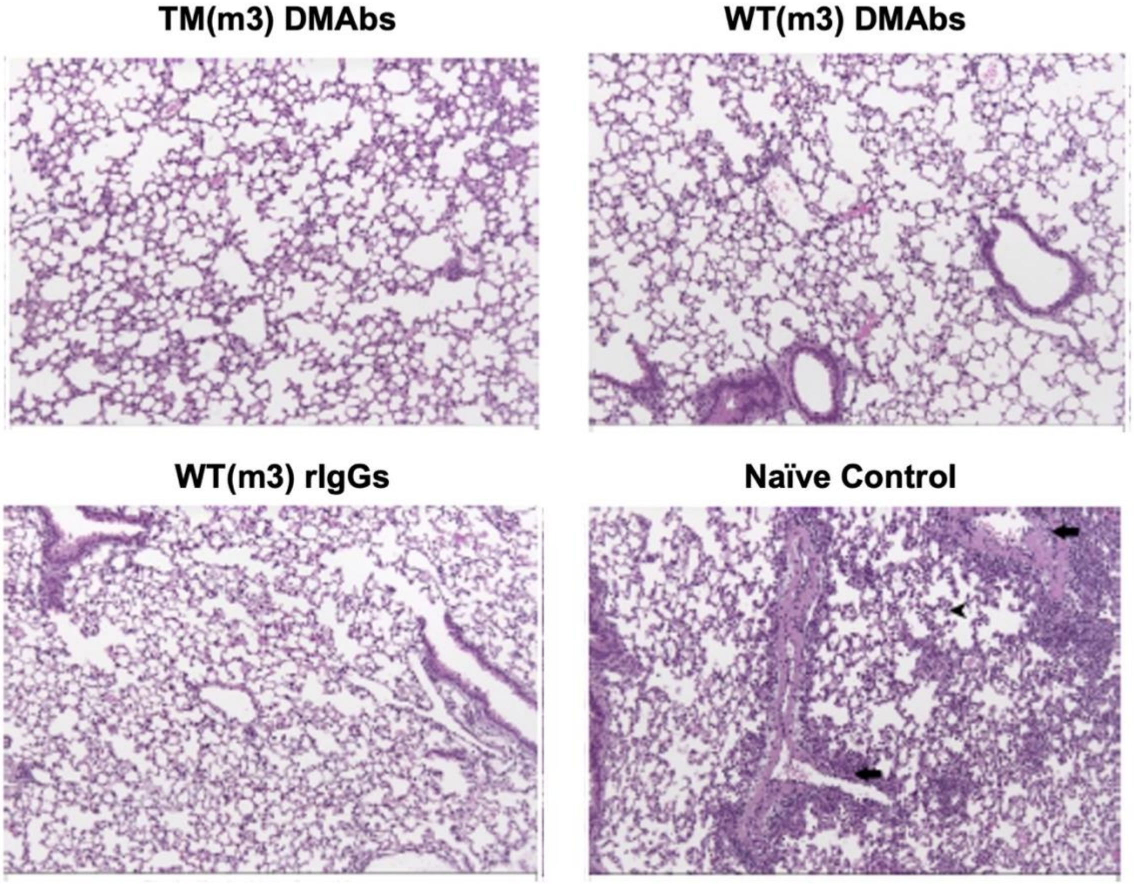

Supplementary Fig. 6. Prophylactic delivery of DMAb or rIgG cocktails prevents lung pathology following SARS-CoV-2 challenge in K-18 mice (*related to Fig. 4e*). Representative images (1 of 4 independent biological replicates/ group) of H&E-stained lung sections (10x magnification) that were assessed for SARS-CoV-2-induced pathology. SARS-CoV-2-related mononuclear cell vascular/perivascular inflammation (arrows) and mononuclear cell alveolar/ interstitial inflammation (arrowhead) were observed in naïve control animals only.

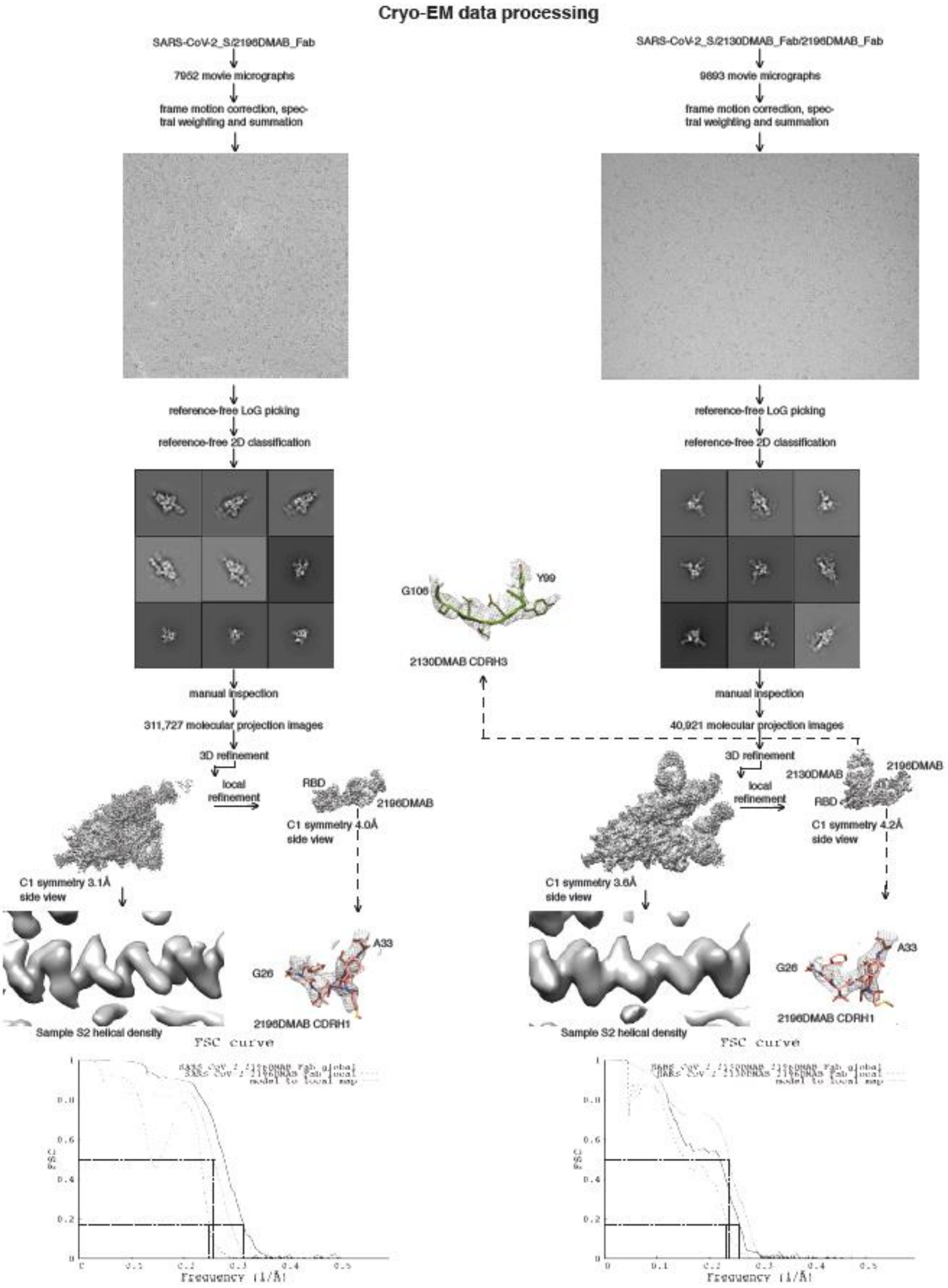

Supplementary Fig. 7. Cryo-EM data processing workflow. Various density segments are displayed for quality assurance. FSC curves for resolution assessment are shown.

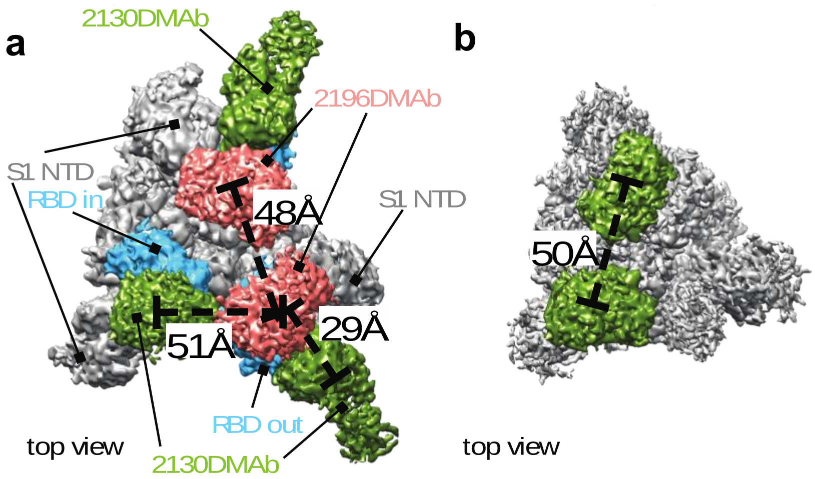

Supplementary Fig. 8. Distance between neighboring dFabs in 2130 and 2196 DMAb complex. a The distance between centers of neighboring 2196 dFabs bound to RBD in 'out' configuration is ~48Å. Distance between neighboring 2130 and 2196 dFabs bound to RBD in 'out' configuration is ~29Å. For the latter, we observe hydrogen bonding between the dFabs in our structure. Taking into consideration the flexibility of this macromolecular complex, it is entirely possible for additional stabilizing interactions to occur between IgGs bound to spike. **b** Model showing the expected distance of ~50Å between centers of 2130 dFabs bound to RBD in 'in' configuration. This distance likewise enables neighboring 2130 IgGs bound to RBD in 'in' configuration to engage in IgG-to-IgG stabilizing interactions.

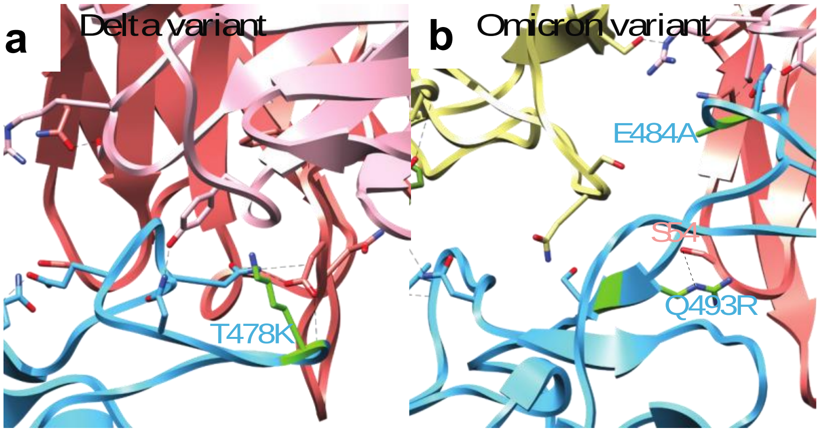

Supplementary Fig. 9. Predictive modeling against B.1.617.2 (Delta) and B.1.1.529/BA.1 (Omicron). **a** The Delta variant exhibits one mutation relevant for 2196 binding (and none for 2130 binding). This T478K Delta mutation is tolerated well; while a hydrogen bond is broken between T478 and 2196 CDRH3 D104, D104 remains hydrogen bonded with RBD. **b** Omicron likewise exhibits the T478K substitution in identical fashion to Delta. In addition, Omicron exhibits Q493R. The hydrogen bond between Q493 and 2196 CDRH2 S54 is replaced by a similar hydrogen bond between R493 and S54. In addition, R493 is in hydrogen bond distance to 2196 CDRH2 N56. Omicron also exhibits E484A. This breaks E484 hydrogen bonding to 2130 CDRL1 S30B; however, the shorter side chain of A484 may hydrophobically pack against 2196 CDRH2 V52-G53.

Supplementary Table 1. Cryo-EM data processing specifications.

Map	SARS-CoV-2_S/ 2196DMAB_Fab	SARS-CoV-2_RBD/ 2196DMAB_Fab	SARS-CoV-2_S/ 2130DMAB_Fab/ 2196DMAB_Fab	SARS-CoV-2_RBD/ 2130DMAB_Fab/ 2196DMAB_Fab
PDB code		8D8R		8D8Q
EMDB code		EMD-27255		EMD-27254
**Data collection**				
Microscope	FEI Titan Krios G4	FEI Titan Krios G4	FEI Titan Krios G4/ FEI Talos Arctica	FEI Titan Krios G4/ FEI Talos Arctica
Voltage (kV)	300	300	300/200	300/200
Detector	Gatan Bioquantum K3	Gatan Bioquantum K3	Gatan Bioquantum K3/FEI Falcon 3	Gatan Bioquantum K3/FEI Falcon 3
Recording mode	Counting	Counting	Counting	Counting
Magnification (incl. post-magnification; EFTEM)	81000/64000	81000/64000	81000/150,000	81000/150,000
Movie micrograph pixelsize (Å)	1.054/0.666	1.054/0.666	1.054/0.97	1.054/0.97
Dose rate (e^-/[(camera pixel)*s])	12.921/17.19	12.921/17.19	12.921/0.94	12.921/0.94
Number of frames per movie micrograph	50	50	50/40	50/40
Frame exposure time (ms)	50	50	50/1000	50/1000
Movie micrograph exposure time (s)	3.99/6.59	3.99/6.59	3.99/40	3.99/40
Total dose (e^-/Å^2)	42.969/59.715	42.969/59.715	42.969/40	42.969/40
Defocus range (µm)	0.4-2.5	0.4-2.5	0.4-2.5	0.4-2.5
**EM data processing**				
Number of movie micrographs	7,952	7,952	9,893	9,893
Number of molecular projection images in map	311,727	311,727	40,921	40,921
Symmetry	C1	C1	C1	C1
Map resolution (FSC 0.143; Å)	3.1	4.0	3.6	4.2
Map sharpening B-factor (Å^2)	-93	-70	-75	-52
**Structure Building and Validation**				
Number of atoms in deposited model		3,325		5,206
RBD		1,550		1,550
2130DMAB VL				884
2130DMAB VH				997
2196DMAB VL		833		833
2196DMAB VH		942		942
glycans		14		14
MolProbity score		0.859 (100%)		0.902 (100%)
Clashscore		0.92		1.57
EMRinger score		3.57		2.29
model to map correlation (FSC 0.5; Å)		3.9		4.2
Deviations from ideal				
Bond length outliers (RMSD; Å, #)		0.011 (0)		0.011 (0)
Bond angles outliers (RMSD; °, #)		1.246 (20)		1.236 (27)
Ramachandran plot				
Favored (%)		97.61		98.33
Allowed (%)		2.39		1.52
Outliers (%)		0.00		0.15
