# Supplementary material for: DNA-delivered antibody cocktail exhibits improved pharmacokinetics and confers prophylactic protection against SARS-CoV-2
Source: Nat Commun. 2022 Oct 6;13:5886. doi: 10.1038/s41467-022-33309-6 (PMC9537531; doi:10.1038/s41467-022-33309-6)
Supplement: Supplementary file 3 — Reporting Summary [file 41467_2022_33309_MOESM3_ESM.pdf]

nature portfolio

Corresponding author(s): David B. Weiner

Last updated by author(s): Aug 28, 2022

Reporting Summary

Nature Portfolio wishes to improve the reproducibility of the work that we publish. This form provides structure for consistency and transparency in reporting. For further information on Nature Portfolio policies, see our Editorial Policies and the Editorial Policy Checklist.

Statistics

For all statistical analyses, confirm that the following items are present in the figure legend, table legend, main text, or Methods section.

n/a	Confirmed	
☐	☒	The exact sample size (*n*) for each experimental group/condition, given as a discrete number and unit of measurement
☐	☒	A statement on whether measurements were taken from distinct samples or whether the same sample was measured repeatedly
☐	☒	The statistical test(s) used AND whether they are one- or two-sided<br>*Only common tests should be described solely by name; describe more complex techniques in the Methods section.*
☒	☐	A description of all covariates tested
☐	☒	A description of any assumptions or corrections, such as tests of normality and adjustment for multiple comparisons
☐	☒	A full description of the statistical parameters including central tendency (e.g. means) or other basic estimates (e.g. regression coefficient) AND variation (e.g. standard deviation) or associated estimates of uncertainty (e.g. confidence intervals)
☐	☒	For null hypothesis testing, the test statistic (e.g. *F*, *t*, *r*) with confidence intervals, effect sizes, degrees of freedom and *P* value noted<br>*Give P values as exact values whenever suitable.*
☒	☐	For Bayesian analysis, information on the choice of priors and Markov chain Monte Carlo settings
☒	☐	For hierarchical and complex designs, identification of the appropriate level for tests and full reporting of outcomes
☒	☐	Estimates of effect sizes (e.g. Cohen's *d*, Pearson's *r*), indicating how they were calculated

Our web collection on statistics for biologists contains articles on many of the points above.

Software and code

Policy information about availability of computer code

Data collection	Instrument software for BioTek Synergy 2 (software version 3.08.01), iOdyssey® CLx Imager (Image Studio/version 5.2.5/ JRE version 1.7.0), Titan Krios G4/ K3 detector, Talos Arctica/ Falcon 3 detector.
Data analysis	Microsoft Excel (version 16.54), GraphPad Prism 9 (version 9.1.2), Relion (version 3.1.2), ctffind4 (version 4.1.14), cryosparc (version 3.3.1), Rosetta antibody application (version 2021.16.61629), Coot (version 0.9.8.3), Rosetta FastRelax (version 2021.16.61629), MolProbity (version 1.19.2), EMRinger (version 1.19.2), Privateer (version MKIV), UCSF Chimera (version 1.15).

For manuscripts utilizing custom algorithms or software that are central to the research but not yet described in published literature, software must be made available to editors and reviewers. We strongly encourage code deposition in a community repository (e.g. GitHub). See the Nature Portfolio guidelines for submitting code & software for further information.

Data

Policy information about availability of data

All manuscripts must include a data availability statement. This statement should provide the following information, where applicable:

- Accession codes, unique identifiers, or web links for publicly available datasets
- A description of any restrictions on data availability
- For clinical datasets or third party data, please ensure that the statement adheres to our policy

Publicly available sequences encoding the variable domains of mAb clones COV2-2196 (PDB: 7L7D), COV2-2130 (PDB: 7L7E) and COV2-2381 (PMID 32651581) were used to generate DMAb constructs. The sequence of SARS-CoV-2 Spike protein from isolate USA/WA-2020 (GenBank Accession MN985325) was used to produce the stabilized trimer for cryo-EM studies. Model building was initiated using Spike RBD from PDB 7E23. Refined structures for S/2196 dFab and S/2196 dFab/ 2130 dFab complexes have been deposited in the PDB under accession numbers 8D8R and 8D8Q, respectively. They were also submitted to EMDB under codes EMD-27255 and EMD-27254, respectively. Source data are provided with this paper.

Human research participants

Policy information about studies involving human research participants and Sex and Gender in Research.

Reporting on sex and gender	These studies do not involve human subjects.
Population characteristics	These studies do not involve human subjects.
Recruitment	These studies do not involve human subjects.
Ethics oversight	These studies do not involve human subjects.

Note that full information on the approval of the study protocol must also be provided in the manuscript.

Field-specific reporting

Please select the one below that is the best fit for your research. If you are not sure, read the appropriate sections before making your selection.

☒ Life sciences ☐ Behavioural & social sciences ☐ Ecological, evolutionary & environmental sciences

For a reference copy of the document with all sections, see nature.com/documents/nr-reporting-summary-flat.pdf

Life sciences study design

All studies must disclose on these points even when the disclosure is negative.

Sample size

No statistical method was used to predetermine sample sizes, which differ based on study design as follows:

1. In vivo/ animal studies:
Individual PK/ expression studies (Fig. 1a-b, e; Fig. 5a) were conducted on 4-5 animals per group, based on our extensive experience with in vivo DMAb delivery small animals (PMID 28935938, 29263874, 30428362, 31697648, 21507511). Larger groups of 13-15 animals were used to determine DMAb titers in the lungs (BAL).

Efficacy studies conducted a lethal K-18 mouse model (BioQual; Figs. 3 and 4a-g) contained 12 animals per group. Based on our previous studies in this challenge model (PMID 35090597) and consistent with the literature (PMID 32839612, 34914544, 35780162, 35062015, 34846168, etc.), this size is sufficient to detect significant differences in SARS-CoV-2 viral load and survival among experimental groups following challenge.

Efficacy study conducted in the AAV6.2FF-hACE-2-transduced BALB/c mouse model (Fig. S4) contained 8 animals per group, as we (PMID 34124612) and others (PMID 32668443) have previously described. This size is sufficient to detect significant differences in SARS-CoV-2 viral load in the lungs of experimental and control mice following challenge.

Efficacy in a Syrian golden hamster model (BioQual; Fig. 4h-n) was evaluated using a group size of 6 animals which is consistent with published literature (PMID 32571934, 35062015).

2. In vitro studies/assays:
Quantification ELISAs (Fig. 1a-b, d-e; Fig. 3b, i; Fig. 4b, i; Supplementary Fig. 4b): Performed on samples from individual animals, resulting in 4-14 biological replicates per group (depending on the animal experiment and sample type) tested in technical duplicate.

Binding ELISAs: Performed on pooled sera (Fig. 1g, Fig. 3p, Fig. 5c-d, Supplementary Fig. 3b; pools contained an equal volume of serum from

	4-5 mice/ group) or individual serum samples (Fig. 3j, containing 12 biological replicates), all tested in technical duplicate.<br><br>ACE-2 Inhibition Assay (Fig. 1h): Performed on individual serum samples, resulting in 5 biological replicates per group tested in technical duplicate.<br><br>SARS-CoV-2 pseudovirus neutralization assays: Performed on group sera pools (Fig. 1c, Fig. 5e-f, pools containing an equal volume of sera from 4-5 mice/ group) or serum samples from individual animals (Fig. 2, Fig. 3q, Fig. 5b, Supplementary Fig. 3c containing 3-5 biological replicates or Supplementary Fig. 4c depicting 2 representative serum samples from each group), tested in technical duplicate to establish reliable neutralization curves.<br><br>SARS-CoV-2 live virus neutralization assays: Performed on group serum pools (Fig. 1f, containing an equal volume of serum from 4-5 mice/ group) or serum samples from individual animals (Fig. 4j containing 6 biological replicates), all tested in technical triplicate to ensure reliable evaluation of CPE.
Data exclusions	No data were excluded.
Replication	In vivo DMAb expression was verified in > 2 independent experiments/ construct conducted in multiple mouse strains (BALB/c, K-18 and/or hFcRn Tg) or hamsters. Expression was successful and consistent in all experiments. YTE- containing DMAbs/ rIgGs were evaluated in a single experiment conducted in in Tg FcRn mice due to the longterm followup.<br><br>A total of five efficacy studies were completed using both lethal (K-18; BioQual) and nonlethal (AAV6.2FF-hACE-2-transduced BALB/c) models as well as hamsters (BioQual). All challenge studies demonstrated complete protection/ survival of DMAb-delivered animals relative to controls.<br><br>Quantitative (Anti-hIgG ELISAs) and functional assays (RBD-binding ELISAs, live and pseudovirus neutralization, receptor-blocking, etc.) were performed on biological samples or recombinantly-produced DMAbs. All samples were tested in duplicate or triplicate, which were similar. In most cases, results were confirmed in > 2 independent experiments.
Randomization	Age and sex matched animals were arbitrarily assigned to experimental groups. In vitro experiments did not require randomization, as these study designs did not involve allocation of samples to different experimental groups.
Blinding	Blinding is not relevant for this study, as this is an observational study.

Reporting for specific materials, systems and methods

We require information from authors about some types of materials, experimental systems and methods used in many studies. Here, indicate whether each material, system or method listed is relevant to your study. If you are not sure if a list item applies to your research, read the appropriate section before selecting a response.

Materials & experimental systems

n/a	Involved in the study	
☐	☒	Antibodies
☐	☒	Eukaryotic cell lines
☒	☐	Palaeontology and archaeology
☐	☒	Animals and other organisms
☒	☐	Clinical data
☒	☐	Dual use research of concern

Methods

n/a	Involved in the study	
☒	☐	ChIP-seq
☒	☐	Flow cytometry
☒	☐	MRI-based neuroimaging

Antibodies

Antibodies used	anti-mouse CD4 (BioXCell; clone GK1.5; cat BE0003-1; 200ug/ dose)<br>anti-mouse CD8 (BioXCell; clone YTS 169.4; cat BE0117; 200ug/ dose)<br>anti-beta actin IgG, produced in mice (Sigma; clone AC-74; cat A5316; 1:5000 dilution)<br>anti-human IgG-IRDye-800CW (LI-COR; cat 926-32232; 1:10000 dilution)<br>anti-mouse IgG- IRDye-680RD (LI-COR; cat 926-68070; 1:10000 dilution)<br>anti-rabbit IgG- IRDye-RD680 (LI-COR; cat 926-68071; 1:10000 dilution)<br>anti-human IgG-Fc (Bethyl; cat A80-104A; 1:200 dilution)<br>anti-human IgG-Fc-HRP (Bethyl; cat A80-104P; 1:10000 dilution)<br>anti-human IgG (h+l)-HRP (Bethyl; cat A80-119P; 1:10000 dilution)<br>Purified human IgG (Bethyl; cat P80-112; 1:2000 dilution)<br>6x-His tag polyclonal antibody (Thermo Fisher; cat PA1-983B; 1:2000 dilution)<br>Anti-YTE IgG (AstraZeneca; clone 23F7.1; 1:5000 dilution)

Validation	Antibodies purchased from commercial vendors were validated by the manufacturers for purity as well as specificity and functionality using a number of molecular techniques including ELISA, immunohistochemistry, western blot, dot blot and/or immunofluorescence. Corresponding certificates of analyses and additional information are available on the product webpages, listed below:<br><br>anti-mouse CD4: https://bxcell.com/product/m-cd4/<br><br>anti-mouse CD8: https://bxcell.com/product/m-cd8/<br><br>anti-beta actin IgG, produced in mice: https://www.sigmaaldrich.com/US/en/product/sigma/a5316<br><br>anti-human IgG-IRDye-800CW: https://www.licor.com/bio/reagents/irdye-800cw-goat-anti-human-igg-secondary-antibody<br><br>anti-mouse IgG- IRDye-680RD: https://www.licor.com/bio/reagents/irdye-680rd-goat-anti-mouse-igg-secondary-antibody<br><br>anti-rabbit IgG- IRDye-RD680: https://www.licor.com/bio/reagents/irdye-680rd-goat-anti-rabbit-igg-secondary-antibody<br><br>anti-human IgG-Fc: https://www.fortislife.com/products/immunoglobulins/goat-anti-human-igg-fc-fragment-antibody/A80-104A<br><br>anti-human IgG-Fc-HRP: https://www.fortislife.com/products/secondary-antibodies/goat-anti-human-igg-fc-fragment-antibody-hrp-conjugated/A80-104P<br><br>anti-human IgG (h+l)-HRP: https://www.fortislife.com/products/secondary-antibodies/goat-anti-human-igg-heavy-and-light-chain-antibody-hrp-conjugated/A80-119P<br><br>Purified human IgG: https://www.fortislife.com/products/antigen-proteins/purified-human-igg-lambda-normal-serum/P80-112#Documents<br><br>6x-His tag polyclonal IgG: https://www.thermofisher.com/antibody/product/6x-His-Tag-Antibody-Polyclonal/PA1-983B<br><br>The anti-YTE mAb was produced at AstraZeneca via hybridoma screening and subsequent chromatography purification. Quantification of monomeric IgG was established by nanodrop (A280) methods and purity was validated by HPSEC. The purified product was verified to be Endotoxin-free.

Eukaryotic cell lines

Policy information about cell lines and Sex and Gender in Research

Cell line source(s)	Expi293F (Thermo Fisher; A14527)<br>HEK293T (ATCC® CRL-3216™)<br>huCHOAce2 cells (Creative Biolabs; VCeL-Wyb019)<br>Vero 536 cells (ATCC®; CCL-81™)
Authentication	All cell lines were purchased from commercial vendors and accompanied by a certificate of analysis.
Mycoplasma contamination	Thermo Fisher and ATCC provide results of mycoplasma testing as part of the certificate of analysis. Mycoplasma testing is periodically conducted to ensure stocks remain negative.
Commonly misidentified lines<br>(See ICLAC register)	No commonly misidentified lines are used in these studies.

Animals and other research organisms

Policy information about studies involving animals; ARRIVE guidelines recommended for reporting animal research, and Sex and Gender in Research

Laboratory animals	Animal Strains:<br>Female BALB/c mice (The Jackson Laboratory), 5-8 wks<br>Female or male K-18 mice (B6.Cg-Tg(K18-ACE2)2Prlmn/J; The Jackson Laboratory), 5-8 wks<br>Female hFcRn mice (B6.Cg-Fcgrttm1DcrTg(FCGRT)32Dcr/DcrJ; The Jackson Laboratory), 5-8 wks<br>Female Syrian golden hamsters (HsdHan:AURA; Envigo); 7-8wks<br><br>Animals were housed at an ambient temperature of 20-23 degrees C and 45-65% relative humidity on a 12hr/12hr light/dark cycle with 15 minute transition periods at dusk and dawn.
Wild animals	These studies did not involve wild animals.

Reporting on sex	DMAb expression and efficacy was confirmed in both male and female animals.
Field-collected samples	These studies did not involve field-collected samples.
Ethics oversight	All animal procedures were conducted in accordance with the following protocols which were approved by the Institutional Animal Care and Use Committees (IACUC) for each study site: 201399 (Wistar), 201464 (Wistar), 20-164 (BioQual), SP2100123 (Inovio).

Note that full information on the approval of the study protocol must also be provided in the manuscript.
